# ISCB’s initial reaction to *New England Journal of Medicine* editorial on data sharing

**DOI:** 10.12688/f1000research.8051.1

**Published:** 2016-02-11

**Authors:** Alfonso Valencia, Scott Markel, Bruno Gaeta, Terry Gaasterland, Thomas Lengauer, Bonnie Berger, Christine Orengo

**Affiliations:** 1 International Society for Computational Biology, Inc. (ISCB), Bethesda, MD, USA

**Keywords:** Data sharing, Data reuse, Data repositories, Data archiving, Open data

## Abstract

This message is a response from the ISCB in light of the recent the *New England Journal of Medicine* (NEJM) editorial around data sharing.

The recent editorial by Drs. Longo and Drazen in the *New England Journal of Medicine* (NEJM) ^1^ has stirred up quite a bit of controversy. As Executive Officers of the International Society of Computational Biology, Inc. (ISCB), we express our deep concern about the restrictive and potentially damaging opinions voiced in this editorial, and while ISCB works to write a detailed response, we felt it necessary to promptly address the editorial with this response. While some of the concerns voiced by the authors of the editorial are worth considering, large parts of the statement purport an obsolete view of hegemony over data that is neither in line with today’s spirit of open access nor further an atmosphere where the potential of data can be fully realized.

ISCB acknowledges that the additional comment on the editorial ^2^ eases some of the polemics, but unfortunately without addressing some of the core issues. We still feel that we need to contrast the opinion voiced in the editorial with what we consider to be the axioms of our scientific society. We feel the following statements would lead to a more fruitful future of data-driven science:

• Data produced with public money should be public for the benefit of science and society
• Restrictions to the use of public data hamper science and slow progress
• Open data is the best way to combat fraud and misinterpretations

Current large data collections proceed from many sources, are continually accumulated, and require a variety of analytical approaches. Data generation and data analysis overlap in time and are continually updated with new data sets produced by new techniques and new analysis methodologies. Furthermore, in many cases current science functions in consortia in which scientists collaborate toward common goals while preserving their own scientific objectives. Dividing scientists into data providers and data analysts is simplistic and gives a misleading impression of the actual state of biological and biomedical science.

ISCB very much supports collaboration between disciplines, including experimental and clinical disciplines as well as bioinformatics, as the best way forward to address complex biological problems. But this collaboration cannot be based on imposed restrictions to data access and cannot be contained in professional silos. (The use of expressions such as “research parasites” clearly does not help.)

In this spirit, ISCB recently launched the ISCB Community Journal (ISCB Comm J), which is published on the F1000Research publishing platform. F1000Research and ISCB Comm J have a robust data sharing policy. All primary research articles include the submission of the data underlying the results, together with details of any software used to process results.

Many bio-communities have made significant progress by endorsing open data policies and, gratefully, public funding agencies have connected to the spirit that they are distributing taxpayers’ money to science and that, therefore, the data that are generated in the course belong to the public. It is, perhaps, natural that some areas of biomedical research are slow in adopting these policies. History and the confidential nature of the relevant data are surely some of the reasons. However, in our opinion, data hegemony is another reason and this has to be overcome. The sooner these barriers to progress are removed the sooner patients will benefit from the current flourishing of biomedical research.
